# Potential role of sympathetic activity on the pathogenesis of massive pulmonary embolism with circulatory shock in rabbits

**DOI:** 10.1186/s12931-019-1069-z

**Published:** 2019-05-22

**Authors:** Yuting Wang, Delong Yu, Yijun Yu, Wusong Zou, Xiaohui Zeng, Liqun Hu, Ye Gu

**Affiliations:** Department of Cardiology, Wuhan Fourth Hospital; Puai Hospital affiliated to Tongji Medical College, Huazhong University of Science and Technology, HanZheng Street 473# QiaoKou District, Wuhan, 430033 China

**Keywords:** Acute pulmonary embolism combined with shock, Pulmonary arterial hypertension, Sympathetic activity, Sodium nitroprusside, Diltiazem

## Abstract

**Background:**

We recently showed that intravenous sodium nitroprusside treatment (SNP) could relieve the pulmonary vasospasm of pulmonary embolism (PE) and non-pulmonary embolism (non-PE) regions in a rabbit massive pulmonary embolism (MPE) model associated with shock. The present study explored the potential role of cardiopulmonary sympathetic activity on the pathogenesis and the impact of vasodilators on cardiopulmonary sympathetic activity in this model.

**Methods:**

Rabbits were randomly divided into sham operation group (S group, *n* = 8), model group (M, equal volume of saline intravenously, *n* = 11), SNP group (3.5 μg/kg/min intravenously, *n* = 10) and diltiazem group (DLZ, 6.0 μg/kg/min intravenously, n = 10).

**Results:**

MPE resulted in reduced mean arterial pressure and increased mean pulmonary arterial pressure as well as reduced PaO_2_ in the M, SNP and DLZ groups. Tyrosine hydroxylase (TH), neuropeptide Y (NPY) and endothelin-1 (ET-1) expression levels were significantly increased, while nitric oxide (NO) levels were reduced in both PE and non-PE regions in the M group. Both SNP and DLZ decreased mean pulmonary arterial pressure, reversed shock status, downregulated the expression of TH, NPY and ET-1, and increased NO levels in PE and non-PE regions.

**Conclusion:**

Present results indicate that upregulation of the sympathetic medium transmitters TH and NPY in whole lung tissues serves one of the pathological features of MPE. The vasodilators SNP and DLZ could relieve pulmonary vasospasm in both embolization and non-embolization regions and reverse circulatory shock, thereby indirectly downregulating the sympathetic activation of the whole lung tissues and breaking a vicious cycle related to sympathetic activation in this model.

## Background

Acute pulmonary embolism (APE) is characterized by respiratory and circulatory dysfunction, which is usually induced by the pulmonary artery and branches emboli from the venous system or right ventricle [1]. Clinical reports indicate that the mortality rate in APE patients with complicating shock, so called massive PE (MPE), is approximately 30% [2]. MPE was also reported to be associated with more than 50% mortality within one hour post admission to hospital [1, 3]. It remains unclear why irreversible respiratory and circulatory failure could still occur in APE patients with embolization area less than 50% [3]. Previous studies showed that enhanced vasoconstrictor substance release was related to cardiopulmonary dysfunction in APE model [4, 5]. Faxon et al. demonstrated that stellate ganglion blockade could improve the clinical symptoms in 3 out of 4 PE patients [6]. Above findings thus suggest that the activation of the sympathetic nervous system might contribute to the pathogenesis of PE. At present, the relationship between the sympathetic nervous system and the pulmonary circulatory failure is not fully understood, and the therapy efficacy remains suboptimal in the case of MPE with complicating shock. In the case of MPE, one therapy option is to use vasodilators aiming to improve pulmonary blood flow [7]. Our previous study showed that continuous intravenous SNP infusion (3.5 μg/kg/min for 120 min) could effectively improve pulmonary blood flow, mitigate hypoxia and reverse shock in the acute MPE [8]. Calcium channel blockers (CCB) were the first vasodilator agents to gain the popular acceptance in the treatment of pulmonary arterial hypertension (PAH). They have been shown to be particularly effective in patients who show a significant immediate haemodynamic response to pulmonary vasodilators (“responders”) [9]. Diltiazem (DLZ) is the mostly used CCBs to relieve pulmonary hypertension, especially in pulmonary hypertensive patients with complicating tachycardia [10]. The role of CCBs, administered intravenously in the acute phase of pulmonary embolism is not yet fully understood. The present study tested the effects of SNP and DLZ in the rabbit model of acute MPE with circulatory shock. Since it was observed that the sympathetic activity was excessively activated in the case of hypoxemia and shock status [5, 11, 12], the effects of SNP and DLZ on the sympathetic activity expression of PE and non-PE tissues are also evaluated.

## Methods

### Animals

All experimental procedures are complied with the Guide for the Care and Use of Laboratory Animals published by the US National Institute of Health (NIH Publication) [13], and approved by the Animal Care Committee of Huazhong University of Science and Technology. Healthy adult New Zealand rabbits, weighing 2.5–3.0 kg, were purchased from the Experimental Animal Center of Tongji Medical College, Huazhong University of Science and Technology. The rabbits were housed under the standard conditions with free access to food and drinking water for 1 week before the main experiments.

### Anaesthesia and custody

Rabbits were anaesthetized by sodium pentobarbital (3%, 20 mg/kg, iv). After the anaesthetization, the rabbit was fixed on the operating table in the supine position and connected to a cardiogram monitor (LEAD-7000, Sichuan Jinjiang Electronic Technology Co. China) for monitoring the heart rate.

### Paracentesis, catheterization and pressure monitoring

The skin of right groin area was prepared and disinfected, and the femoral artery and femoral vein were isolated. The Seldinger puncturing method was used to puncture the femoral artery and the femoral vein, and 5F sheath tubes (Radio focus TERUMO) were then placed. Under the X-ray guidance from the perspective projection view, 4F Cordis catheter (Cordis Corporation, Florida, USA) was inserted into the main trunk of the pulmonary artery through the femoral vein. The 5F sheath inserted to the femoral artery and the tail end of the 4F Cordis catheter were connected to the pressure transducer for real-time continuous monitoring of the mean arterial pressure (MAP) and mean pulmonary artery pressure (MPAP) by LEAD-7000 monitor (Sichuan Jinjiang Electronic Technology Co. China).

### Establishment of MPE model

The MPE model used in this study was established as previously described [8]. Briefly, the autologous blood clots were prepared to form the emboli according to the methods described in previous studies [14–16]. The autologous blood clots were then injected into the main pulmonary artery of rabbit to establish the MPE model [8]. The model was considered successful when reaching both of the following criteria for 2 min: a) The MAP reduced to below 60 mmHg and b) the MPAP increased to up to 2 times of the baseline level. MAP and MPAP were dynamically monitored during this period.

### Groups and drug administration

Thirty-nine rabbits were used. 8 rabbits underwent sham operation (S group, puncture and catheterization, bolus injection of saline to the main pulmonary artery). Thirty-one rabbits were used to establish the MPE model, and all survived. 11 rabbits were randomly assigned to the model (M) group (puncture and catheterization, bolus injection of autologous blood clots until the shock status was reached, followed by equal volume of saline iv. for 120 min), 10 rabbits were assigned to the SNP group (puncture and catheterization, bolus injection of autologous blood clots until the shock status was reached, followed by SNP iv. 3.5 μg/kg/min for 120 min), and 10 rabbits were assigned to the diltiazem (DLZ) group (puncture and catheterization, bolus injection of autologous blood clots until the shock status was reached, followed by DLZ iv. 6.0 μg/kg/min for 120 min).

### Haemodynamic changes and arterial blood gas analysis

The MAP and MPAP were real-time continuously monitored by LEAD-7000 monitor (Sichuan Jinjiang Electronic Technology Co. China). In addition, blood samples were collected before embolization, immediately after shock status was reached, and at the end of the experiment for the blood gas analysis (GEM Premier 3000, Instrumentation Laboratory Co. America); pH, PaCO_2_ and PaO_2_ values were compared at various time points among groups.

### Lung tissue collection and biochemical analysis

All rabbits received an intravenous injection of 10 mL 10% potassium chloride for euthanasia, and then the whole lung tissues were excised for postmortem examination. The pulmonary vessels were longitudinally dissected to examine the positions of injected thromboembolus. The embolized and non-embolized lung tissues were identified according to the gross anatomical specimen evaluation, and the relevant tissues were taken and washed with 25 U/mL heparin. The embolized and non-embolized lung tissues were subsequently homogenized. The levels of ET-1 (ab133030, Abcam, Cambridge, MA, USA) and NO (Nanjing Jiancheng Bioengineering Institute, Nanjing, China) in embolized and non-embolized areas were determined by ELISA method.

### Pathology examination

Immunohistochemical staining was performed on lung tissue sections to analyse sympathetic nervous activity with the following antibodies: tyrosine hydroxylase (TH) (1:300, Abcam, Cambridge, Massachusetts, USA) and neuropeptide Y (NPY) (1:500, Abcam, Cambridge, Massachusetts, USA). Immunoreactivity was quantified using the commercial software (Image Pro Plus, Media Cybernetics, Inc., Rockville, Maryland, USA), and evaluated by assessing the percent of positive stained area of the sections.

### Western blotting

Total proteins were extracted from lung tissues with the radio immunoprecipitation assay (RIPA) buffer. Total protein concentration was determined by the bicinchoninic acid (BCA) method. After electrophoresis (SDS-PAGE), membrane transfer and blocking (milk protein), the target membranes were incubated with following primary antibodies at 4 °C overnight: anti-TH polyclonal antibody (1:200, Abcam, Cambridge, Massachusetts), anti-NPY polyclonal antibody (1:1000, Abcam, Cambridge, Massachusetts). The chemiluminescence method was performed to obtain the immunoreactive bands after probing was performed with the secondary antibody (Goat-anti-rabbit, Goat-anti-mouse, KPL, USA). Images were captured and semi-quantitatively analysed by Quantity One (Bio-Rad, USA).

### Statistical analysis

All statistical data are expressed as mean ± SD. All data were first evaluated for normal distribution using Shapiro-Wilk test. Haemodynamic and blood gas parameters were compared by 2-way ANOVA followed by Tukey’s test. One-way ANOVA followed by Tukey’s or Games–Howell’s post hoc test was used to evaluate the differences among the four groups. The Kruskal-Wallis non-parametric test was employed to analyse non-normal distribution variables. The mortality rate in the SNP, DLZ and M groups were compared by log rank test of Kaplan-Meier curves. The value of *P* < 0.05 was considered statistically significant. All statistical analyses were performed with IBM SPSS 19.0 software.

## Results

### Survival results

**Fig. 1:**
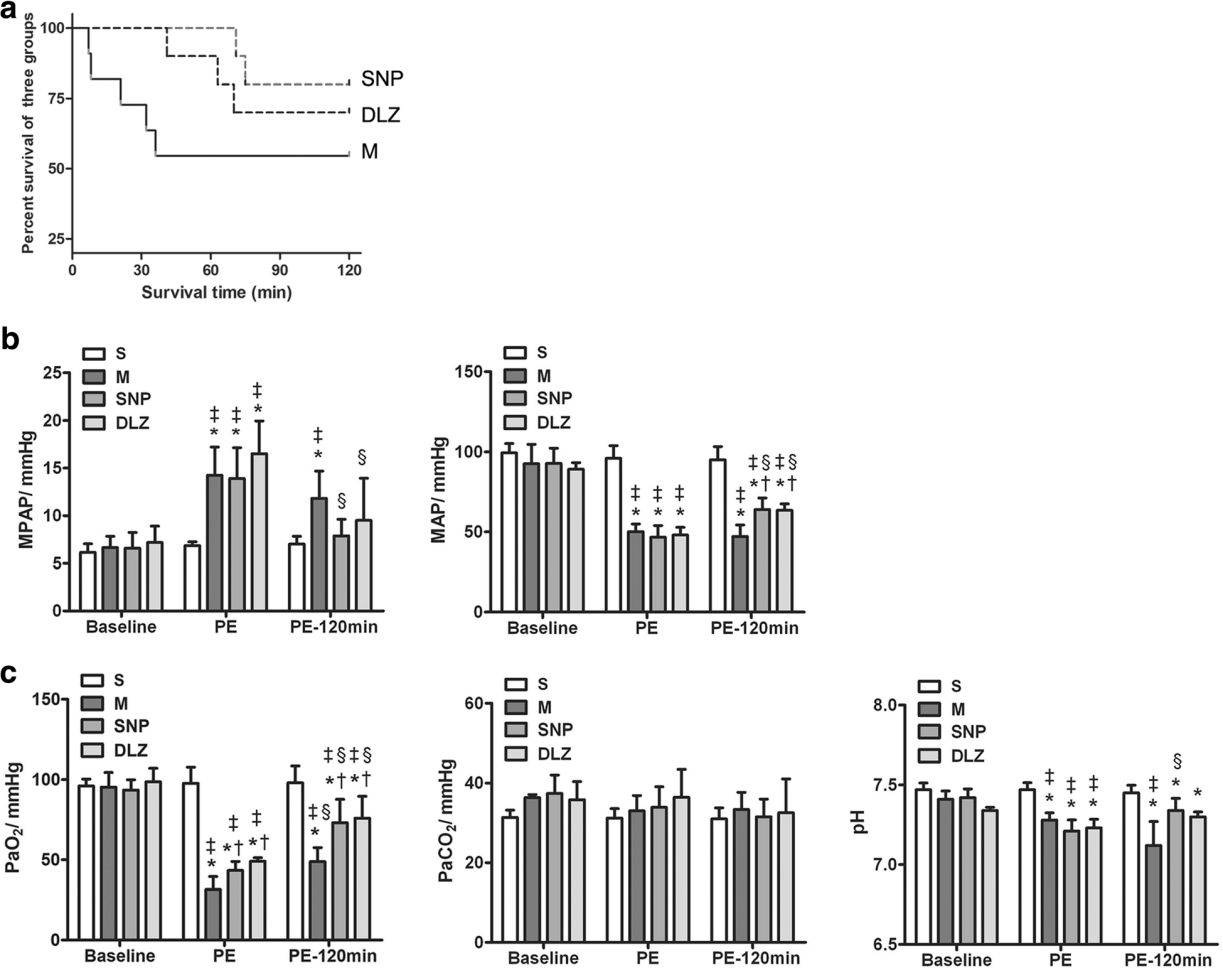
Kaplan-Meier survival curves (**a**), mean pulmonary artery pressure (MPAP) and mean arterial pressure (MAP) changes (**b**) and blood gas analysis (**c**) among various groups. * *P* < 0.05 versus S group, † *P* < 0.05 versus M group. ‡ *P* < 0.05 versus Baseline, § *P* < 0.05 versus PE. S, sham operation group; M, model group; SNP, sodium nitroprusside group, DLZ, diltiazem group. Baseline, before pulmonary embolism; PE, pulmonary embolism referring to the moment of shock status post bolus injection of autologous blood clots; PE-120 min, at 120 min after the initiation of treatment

All 8 rabbits in the S group, 6 out of 11 rabbits in the M group, 8 out of 10 rabbits in the SNP group and 7 out of 10 rabbits in the DLZ group survived to the end of the study. The mortality rate was similar among the SNP, DLZ and M groups (*P* = 0.267, Fig. 1a). MAP, PaO_2_ and PaCO_2_ were lower than 30 mmHg, while pH was below 7.20 in all died rabbits, indicating that the irreversible shock and hypoxic acidosis might be the reasons for death.

### Haemodynamic and blood gas results in all of the rabbits

Figure 1b showed that the MPAP levels were similar at baseline among all groups (*P* > 0.05), significantly increased in the M, SNP and DLZ groups at the shock status induced by bolus injection of autologous blood clots, and were significantly lower in the SNP and DLZ groups than in the M group at 120 min after therapy. Moreover, MAP was significantly reduced in the M, SNP and DLZ groups at the shock status induced by bolus injection of autologous blood clots and MAP was significantly higher in the SNP and DLZ groups than in the M group at 120 min after therapy (Fig. 1b).

Blood gas results were similar among the groups at baseline. PaO_2_ and pH (Fig. 1c) were significantly lower in the M, SNP and DLZ groups than in the S group (all *P* < 0.05), indicating a state of respiratory failure at the shock status induced by bolus injection of autologous blood clots. PaO_2_ and pH were significantly higher in the SNP and DLZ groups than in the M group at 120 min after therapy (P < 0.05).

### Gross morphology of the lungs

**Fig. 2:**
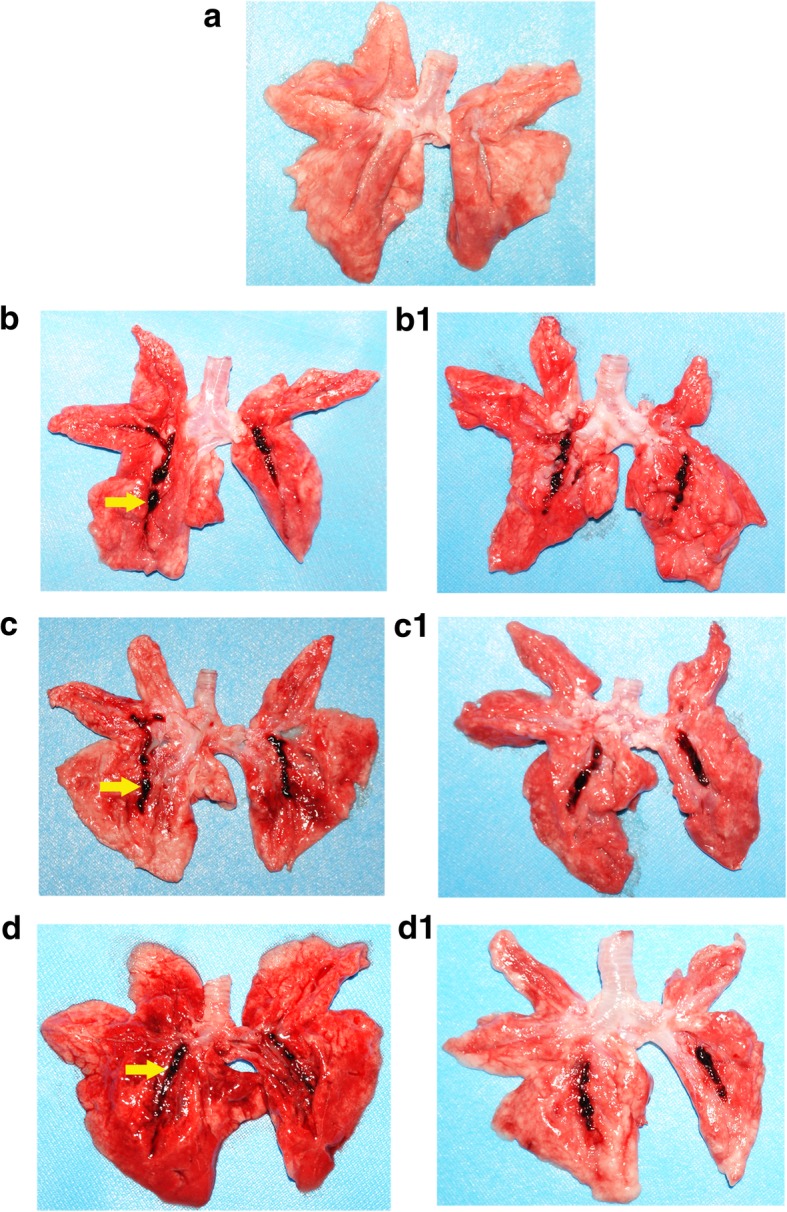
The gross anatomical morphology of the representative lungs. Histological features in the (**a**) S, (**b**) M, (**c**) SNP and (**d**) DLZ groups at the scheduled study end. Histological features of rabbits immediately after death before scheduled study end: b1 (M), c1 (SNP) and d1 (DLZ). S, sham operation group; M, model group; SNP, sodium nitroprusside group; DLZ, diltiazem group. Yellow arrows indicate the autologous thrombus clot

Representative gross morphologies of the lung at scheduled study end from the four groups and lung tissue obtained immediately after death prior the scheduled study end were displayed in Fig. 2. The injected autologous blood clot (arrow) was observed in the pulmonary vascular trunks of the left and right lower pulmonary lobes in the M, SNP and DLZ groups. The left and right upper lobes had no embolism and were thus regarded as non-PE section. The left and right lower lobes were blocked with blood clots, and defined as PE areas.

### Sympathetic neurotransmitter expression in the lung tissues

**Fig. 3:**
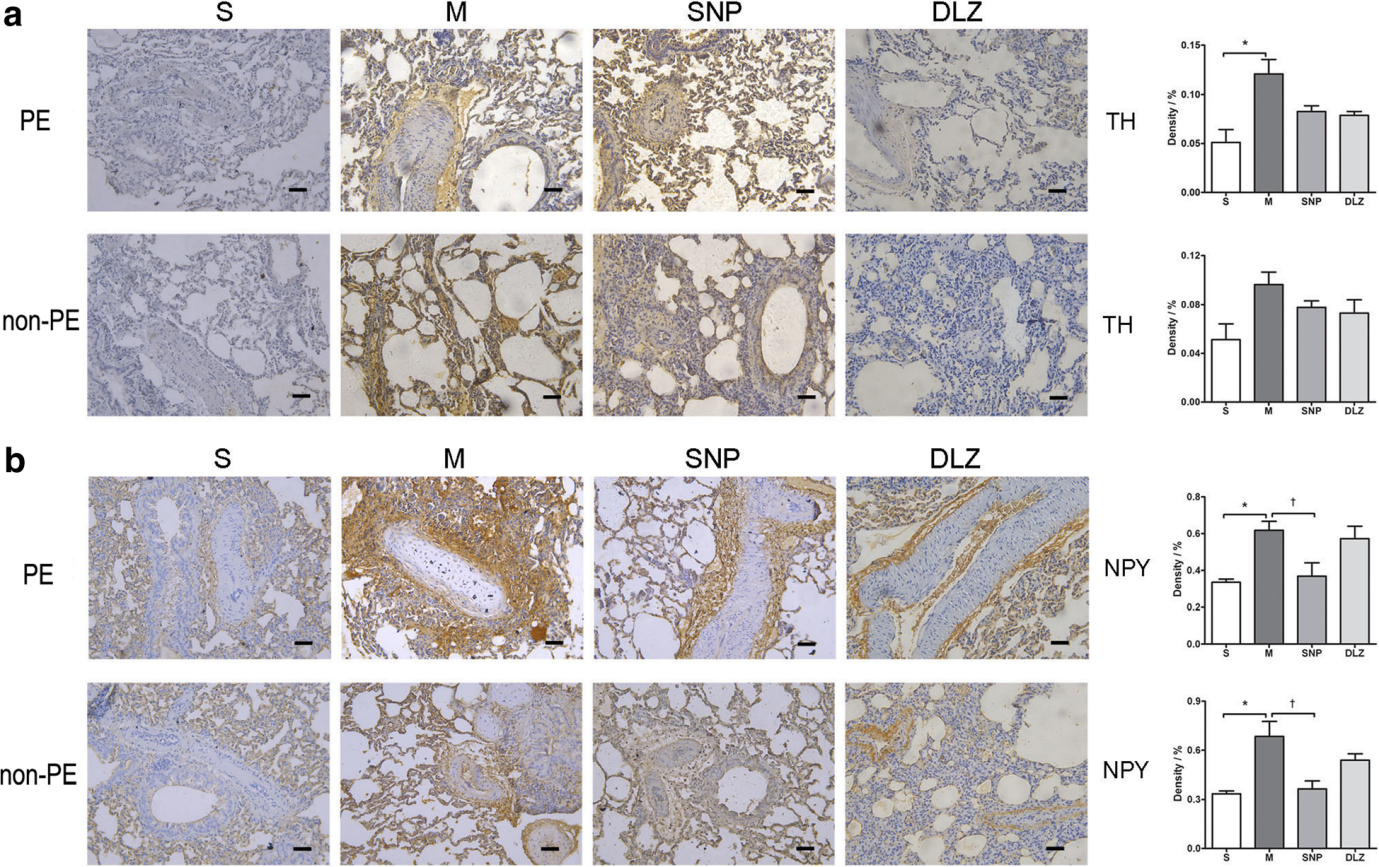
The expression of TH (**a**) and NPY (**b**) in PE and non-PE areas. Data are expressed as mean ± SD by bar graphs. * *P* < 0.05 versus S group, † *P* < 0.05 versus M group. S, sham operation group; M, model group; SNP, sodium nitroprusside group; DLZ, diltiazem group. PE, pulmonary embolism tissue; non-PE, non-pulmonary embolism tissue. Scale bars =50 μm. Original magnification × 200

TH and NPY are sympathetic nervous system activity markers. As indicated in Fig. 3a and Fig. 3b, TH and NPY expression in PE tissues was significantly upregulated in the M group as compared to the S group (*P* < 0.05). The level of NPY in non-PE tissues was also significantly higher in the M group than in the S group (*P* < 0.05). TH expression in PE and non-PE tissues tended to be lower in the SNP and DLZ groups as compared with the M group, NPY expression in PE and non-PE tissues was significantly downregulated in the SNP group (P < 0.05) and tended to be lower in the DLZ group in comparison with the M group.

**Fig. 4:**
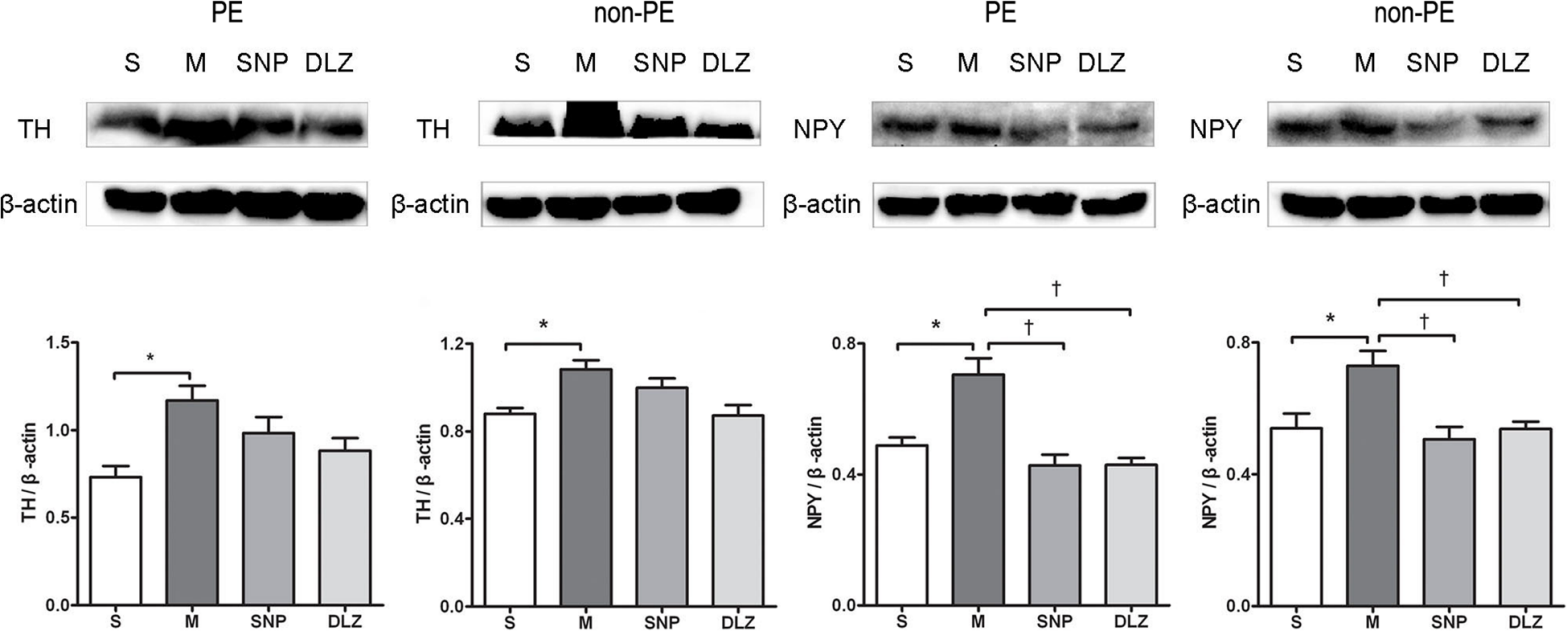
Protein expressions of TH and NPY in PE and non-PE tissues detected by Western-blot. Data are expressed as mean ± SD by bar graphs. * *P* < 0.05 versus S group, † *P* < 0.05 versus M group. S, sham operation group; M, model group; SNP, sodium nitroprusside group; DLZ, diltiazem group. PE, pulmonary embolism tissue; non-PE, non-pulmonary embolism tissue

Western-blot results (Fig. 4) demonstrated that TH and NPY expression in PE and non-PE tissues was significantly higher in the M group than in the S group. TH expression in PE and non-PE tissues tended to be lower in the SNP and DLZ groups, and NPY expression in PE and non-PE tissues was significantly downregulated post the SNP and DLZ treatments (*P* < 0.05).

### ET-1 and NO levels in lung tissues

As shown in Table 1, ET-1 level was significantly increased, while NO was reduced in non-PE tissue in the M group, ET-1 level in non-PE and PE regions was significantly lower in the SNP and DLZ groups than in the M group (P < 0.05). NO level in non-PE and PE regions was significantly higher in the SNP group than in the M group (P < 0.05).

**Table 1:** The contents of ET-1 and NO in lung tissues

Parameters	Tissues	S	M	SNP	DLZ
ET-1 (pg/ml)	PE non-PE	8.42 ± 0.925 8.28 ± 0.944	10.30 ± 1.353 10.77 ± 1.287^*^	7.03 ± 1.486^**†**^ 7.34 ± 1.264^**†**^	7.91 ± 1.019^**†**^ 7.54 ± 0.543^**†**^
NO (μmol/prot)	PE non-PE	0.28 ± 0.069 0.27 ± 0.073	0.17 ± 0.031 0.14 ± 0.067^*^	0.51 ± 0.155^*†^ 0.35 ± 0.066^**†**^	0.28 ± 0.101 0.24 ± 0.075

Data are expressed as means±SD

* *P* < 0.05 versus S group, † *P* < 0.05 versus M group

ET-1, endothelin-1; NO, nitric oxide

*S* sham operation group, *M* model group, *SNP* sodium nitroprusside group, *DLZ* diltiazem group

*PE* pulmonary embolism tissue; non-PE, none pulmonary embolism tissue

## Discussion

**Fig. 5:**
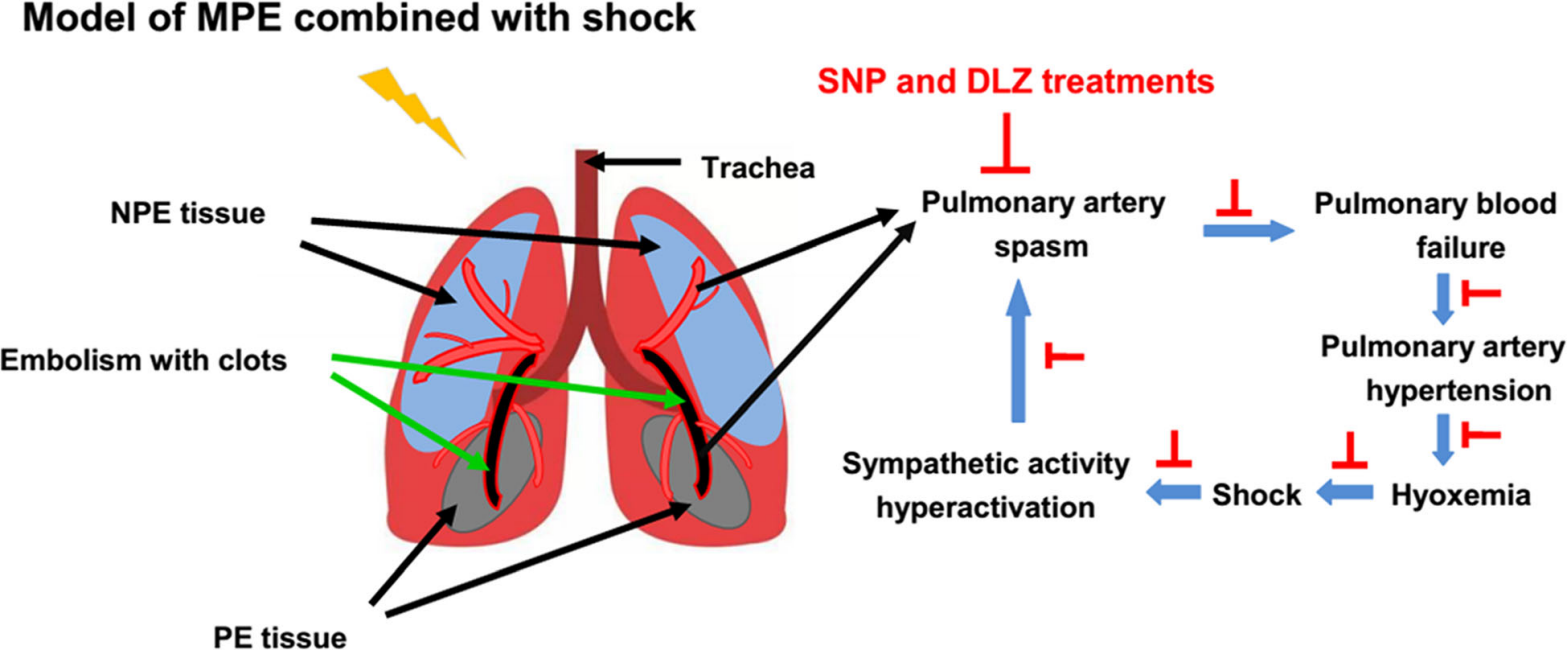
Pathological changes of MPE and potential working effects of SNP and DLZ

The major findings of the present study were as follows: In addition to SNP, DLZ was also effective in mitigated hypoxia and haemodynamic status in this rabbit MPE model. TH, NPY and ET-1 expressions in non-PE and PE regions were increased, while NO was reduced in the M group. Treatment with SNP or DLZ not only mitigated the hypoxia and haemodynamics, but also reduced the sympathetic activation in lung tissues in this MPE model (Fig. 5). These results indicate that the upregulation of the sympathetic activity in PE and non-PE tissues might further aggravate the pulmonary artery spasm and reduce the pulmonary blood flow in this rabbit MPE model [8]. Intravenous SNP and DLZ could dilate the pulmonary vessels and thereby relieve the pulmonary artery spasm in both PE and non-PE regions, thus improve the pulmonary blood flow and increase the blood filling to the left ventricle and restore the systemic circulation. The above changes might also be paralleled by reduced stimulus to systemic pressure/chemoreceptors, and thereby reduce the signaling input transmitted to the central sympathetic nervous system. As a whole, sympathetic activation could be indirectly reduced in this MPE model of rabbits post the intravenous SNP and DLZ therapy, and a vicious cycle of pulmonary vasculature spasm due to excessive sympathetic activation post-PE could be lessened in this rabbit MPE model.

### Sympathetic activity in PE and non-PE tissues of MPE rabbits

It has been observed that the rapid increased pulmonary arterial pressure and increased pulmonary vascular resistance could reduce the cardiac output due to the reduction of pulmonary blood flow and subsequent filling of the left ventricle during acute MPE [8]. In our previous study [8], vasospasm was evidenced both in the PE and non-PE regions. This phenomenon might partly explain why embolization in less than 50% of the lung area could induce systemic shock and death in APE patients [3].

It is known that under the scenario of the shock and hypoxia, the systemic pressure/chemoreceptors would be activated, which could increase the sympathetic signaling input to the central sympathetic nervous system, and result in the activation of sympathetic nervous system [17]. This process would further aggravate vasoconstriction of pulmonary arteries, thus forming a vicious cycle and aggravating shock and hypoxemia in the setting of acute MPE [5, 18]. Previous studies demonstrated that the hypoxemia, shock and pulmonary hypertension could induce the hyperactivation of the sympathetic response [5, 19, 20]. In addition to the direct effects of the sympathetic nerve activity, the imbalance of vasoconstriction/vasodilation mediators (ET-1 and NO) might also be involved in the pulmonary circulatory failure in MPE. Accordingly, the present study showed that ET-1 was increased and NO level was reduced in the lung tissues in this MPE model. Taken together, these data indicate that increased sympathetic activity in both PE and non-PE tissues belong to the pathological features in the acute MPE, which might contribute to the vicious cycle of hypoxemia, shock and sympathetic hyperactivity observed in this MPE model.

### Intravenous SNP and DLZ therapy indirectly downregulated sympathetic activity in lung tissues

The above findings indicate that it is essential to attenuate pulmonary artery spasm in both non-PE and PE regions during the acute phase of MPE to improve shock in the case of acute MPE. Attenuation of sympathetic activity in the lung tissue surely contributes to the relief of pulmonary artery spasm in the case of MPE. Related guidelines recommend thrombolysis therapy as an important first-line therapeutic option in patients with APE [21]. Our present work tested the efficacy of SNP and DLZ for the treatment of MPE in the absence of thrombolysis agents.

Theoretically, the peripheral blood pressure could be further reduced by the use of vasodilating drugs in shock status, which was the potential risk of using vasodilators in this MPE model. However, our experimental results showed that SNP and DLZ attenuated the pulmonary artery spasm, reduced the pulmonary hypertension, decreased the sympathetic activity in the lung tissues, restored the peripheral circulation and improved the haemodynamic status in this rabbit MPE model. The underlying reasons might be explained as follows. Reversing the attenuated pulmonary blood flow by dilating the pulmonary artery with vasodilators might in fact result in the improvement of systemic haemodynamics in this MPE model of rabbits, indicating intravenous vasodilator therapy might be a potential therapy option for the acute MPE.

The key for successful handling of the MPE by vasodilators might be that we should ideally achieve the selective dilatation of the pulmonary artery without lowering the systemic blood pressure. Studies have shown that the antihypertensive effects of SNP and DLZ might differ depending on the drug doses [22, 23]. Our study demonstrated that the intravenous SNP and DLZ at the used dosage in this study improved the pulmonary blood flow, reduced the pulmonary artery pressure and reversed the systemic circulation, indicating that the pulmonary dilating effect of SNP and DLZ was far greater than the blood pressure-lowering effects on the peripheral circulation in this MPE model. This phenomenon could be explained by the following rationale: When SNP/DLZ entered the pulmonary circulation, the effective drug concentration could relieve the pulmonary artery spasm and the pulmonary blood flow could thereby be restored. The restoration of pulmonary blood flow might result in increased blood filling in the left ventricle, thereby mitigating hypoxemia and attenuating shock status in acute MPE rabbits. As the vascular bed of the systemic circulation is much larger than that of the pulmonary circulation, the treatment concentration of SNP and DLZ, that is effective in the lung, might only result in the negligible effects in the peripheral circulation.

The present study explored the effects of vasodilator treatment on the sympathetic activity in the rabbit MPE model. We observed that SNP and DLZ treatments reduced the expression of sympathetic mediators in the lung tissue, both in the PE and non-PE regions. In our view, this effect contributed significantly on lessening the vicious cycle of hypoxemia, pulmonary vasculature spasm and sympathetic activation [5, 12]. It is to note that the observed reduced sympathetic activation post SNP and DLZ might not be a specific therapeutic effect of SNP or DLZ on the sympathetic mediators. The achieved results could rather be viewed as an indirect consequence of reduced sympathetic signalling input to the central sympathetic nervous system by systemic pressure/chemoreceptors, followed by increased pulmonary flow and increased blood filling in the left ventricle in SNP and DLZ treated animals [19, 20]. The mitigation of the hypoxemia and shock observed post-SNP and post-DLZ might be viewed as the key factor responsible for the negative feedback regulation of pressure receptors /chemoreceptors, and reduced the excitability of sympathetic neurons, thus indirectly responsible for the reduced sympathetic activity [19, 20]. Reducing the sympathetic activation might contribute to lessening the vicious cycle of the ominous combination of hypoxemia, shock and sympathetic hyperactivation observed in acute MPE. The above mentioned attenuated sympathetic activity might be an essential element responsible for the observed final net beneficial effect of SNP and DLZ on systemic circulation in this MPE model.

### Changes of sympathetic and parasympathetic nervous system activity in MPE

Recent study indicated that both the sympathetic and parasympathetic nervous systems played an important role in maintaining the sympathovagal balance in normal homeostasis [24]. The sympathovagal imbalance is a known reason of cardiac dysfunction [24] and abnormal vascular dynamics [25]. It is possible that the decreased parasympathetic activity might also contribute to the observed sympathetic activity enhancement in this MPE model. Due to inexperience, the parasympathetic expression was not examined in this study and further studies are warranted to explore the role of parasympathetic activity in this model as well as the change post intravenous SNP and DLZ therapy.

### Study limitations

There are several study limitations in our present experiment.

1. The catecholamine substance such as norepinephrine (NE) in the lung tissues was not determined in our study. The reasons were as follows: 1) We could not purchase NE ELISA kit (for rabbit species). 2) NE is unstable and easy to degrade in the isolated tissue, and thus needs to be detected immediately. However, it was difficult to finish the rapid NE detection in lung tissues obtained from multiple rabbits in our laboratory. As the rate limiting enzyme of catecholamine synthesis, TH has good stability and could indirectly reflect the level of neurotransmitter synthesis [26]. Thus, TH was detected in rabbit pulmonary tissues as an indirect indicator of the sympathetic activity of lung tissues in the present study.
2. We have no data on the effects of directly blocking the sympathetic nerve activity in this model. It remains unknown whether blocking TH, NPY and ET-1 would achieve comparable effects in this MPE rabbit model, and future studies are needed to address related issues. Experiments to lessen the sympathetic nerve activity by cutting T1-T6 sympathetic ganglia, as reported in a previous study [27], in this rabbit model of MPE are needed to clarify this issue.
3. Parasympathetic markers were not determined in this MPE model, so the parasympathetic nervous system (PNS) activity and its change post SNP and DLZ therapy in this model remains unknown now. Future studies are warranted to explore the role of PNS in this model and PNS changes post SNP and DLZ therapy through measuring related markers such as acetycholine.

## Conclusions

In conclusion, the upregulation of sympathetic activity in the whole lung tissue may aggravate the pulmonary artery spasm in PE and non-PE regions, thus forming a vicious cycle of the pulmonary hypertension, peripheral circulatory failure and sympathetic activation in the setting of MPE. By controllable intravenous drip, the vasodilators, SNP and DLZ, could relieve the pulmonary artery spasm, restore the pulmonary blood flow and improve the peripheral circulation. Moreover, the use of the vasodilators SNP and DLZ via the applied route and dosage is associated with reduced sympathetic activity in both PE and non-PE regions in this MPE model, and these effects are helpful for maintaining the net beneficial effects of SNP and DLZ in this rabbit MPE model.

## Abbreviations

APE: Acute pulmonary embolism
DLZ: Diltiazem
ET-1: Endothelin-1
M: Model
MAP: Mean arterial pressure
MPAP: Mean pulmonary artery pressure
MPE: Massive pulmonary embolism
NE: Norepinephrine
NO: Nitric oxide
non-PE: non-pulmonary embolism
NPY: Neuropeptide Y
PaO_2_: Oxygen pressure
PE: Pulmonary embolism
S: Sham
SNP: Sodium nitroprusside
TH: Tyrosine hydroxylase
